# Behavioral Determinants in Pediatric Dentistry: A Comparative Analysis of Cooperative Versus Uncooperative Patients

**DOI:** 10.3390/children13040516

**Published:** 2026-04-08

**Authors:** Narmin Helal, Nisma Merdad, Heba Jafar Sabbagh

**Affiliations:** 1 Pediatric Dentistry Department, Faculty of Dentistry, King Abdulaziz University, Jeddah 21589, Saudi Arabia; hsabbagh@kau.edu.sa; 2 Department of Clinical Psychology, Effat University, Jeddah 22332, Saudi Arabia; nmerdad@effatuniversity.edu.sa

**Keywords:** dental anxiety, patient cooperation, pediatric dentistry, parent–child relations

## Abstract

**Highlights:**

**What are the main findings?**

• Fear of losing control demonstrated the strongest association with child behavior in pediatric dental patients, with children reporting this fear showing significantly lower odds of cooperative behavior;
• Maternal dental anxiety was significantly associated with child behavior, with higher maternal anxiety linked to reduced odds of cooperation;
• Demographic variables, including age, gender, and income, were not significantly associated with cooperative behavior.

**What are the implications of the main findings?**

• Pediatric dental behavior management strategies should incorporate approaches that enhance children’s perceived control during treatment, as control-related cognitions appear to play a central role in behavioral responses;
• Screening for and addressing maternal dental anxiety may contribute to improved behavioral outcomes during pediatric dental visits;
• Observable cooperation should not be interpreted as an absence of anxiety; children who appear behaviorally compliant may still experience underlying cognitive or emotional distress related to perceived lack of control.

**Abstract:**

**Background/Objectives**: Uncooperative behavior in pediatric dental settings remains a significant barrier to effective treatment. Factors such as demographics, psychological variables, and family influences may impact children’s behavior, but their relative importance is not fully understood. This study explores the emotional, familial, and demographic factors influencing cooperation among children in dental clinics in Jeddah, Saudi Arabia. **Methods:** A cross-sectional study was conducted among children aged 6–11 years attending dental clinics in Jeddah, Saudi Arabia. Participants undergoing non-invasive dental procedures were recruited. Behavioral cooperation was assessed using the Frankl Behavior Rating Scale, and dental anxiety was measured using the validated Abeer Children Dental Anxiety Scale (ACDAS). Data on demographic characteristics, child cognitive factors, and parental dental anxiety were collected through structured interviews. Multivariable logistic regression analysis was performed to identify independent determinants of cooperative behavior. **Results:** A total of 906 children were included in the analysis. Demographic variables, including gender, age, and income, were not significantly associated with child behavior (all *p* > 0.05). Fear of losing control emerged as the strongest predictor in the model. Children reporting fear of losing control had significantly lower odds of cooperative behavior (AOR = 0.14, 95% CI [0.10–0.22], *p* < 0.001). Shyness in the clinic was not statistically significant (*p* = 0.216). Maternal dental anxiety was significantly associated with child behavior, with higher maternal anxiety scores linked to lower odds of cooperative behavior (AOR = 0.96, 95% CI [0.93–0.997], *p* = 0.032). Paternal dental anxiety was not significantly associated with child behavior (*p* = 0.701). **Conclusions:** Fear of losing control and maternal dental anxiety were independently associated with children’s behavioral responses during dental visits. These findings highlight the relevance of children’s perceived control and maternal anxiety in understanding behavioral outcomes in pediatric dental settings.

## 1. Introduction

Cooperation during pediatric dental visits is crucial for successful treatment, patient comfort, and long-term oral health. Uncooperative behavior can pose challenges, leading to longer procedures, increased stress for both patient and dentist, and potential future avoidance of dental care [1]. While some children adapt quickly to dental settings, others resist, avoid, or become distressed, highlighting the importance of understanding what promotes cooperation [2]. Research indicates that cognitive and emotional factors greatly influence a child’s willingness to cooperate. Cognitively, differences in perception, communication, and self-awareness can affect how children interpret and respond during dental visits [3]. Emotionally, dental anxiety, whether felt by the child or parent, plays a significant role, as children often mirror their parents’ fears [4]. In Saudi Arabia, pediatric dental anxiety seems common, yet there is limited research on the specific cognitive and emotional factors that predict cooperation among Saudi children [5]. Dental anxiety is a key barrier to cooperation during pediatric dental visits. Studies show that anxious children often behave uncooperatively, from crying to refusal [6]. In Saudi Arabia, nearly 45% of children experience dental fear, often linked to pain, negative experiences, or unfamiliarity [7,8]. Parental influence worsens this, as children learn behaviors through observing anxious parents [9], with Saudi studies confirming higher anxiety and uncooperative behavior among children of anxious parents [5]. Prior positive dental experiences improve cooperation by reducing fear and building confidence [1], while negative or absent early experiences increase anxiety and avoidance [6]. In Saudi Arabia, insufficient early preventive care likely contributes to higher dental anxiety and lower cooperation [10]. Addressing dental anxiety in children and parents, alongside positive early experiences, is crucial for improving cooperation.

Self-consciousness about dental aesthetics describes the extent to which children worry about the appearance of their teeth and how others may judge them. Research from Saudi Arabia indicates that children with obvious dental malocclusion or decay are more susceptible to social wellbeing problems, which can in turn fuel uncooperative conduct in the dental chair [11]. This reluctance is frequently tied to apprehensions about being judged, diminished self-esteem, or previous adverse social encounters related to the look of their teeth [12]. Concerns related to dental appearance and self-perception may contribute to heightened distress and avoidance behaviors in clinical settings, particularly among children with visible dental problems [11,12].

Fear of losing control is strongly linked to uncooperative behavior. The Cognitive-Affective Model of Dental Anxiety states that perceptions of uncontrollability in dental settings increase distress and likelihood of avoidance [13]. Children’s dental fear often involves feelings of uncontrollability and unpredictability, influencing their responses during treatment [7]. This fear worsens when children feel a lack of autonomy, leaving them trapped or powerless [14]. In Saudi Arabia, cognitive and emotional factors like fear, anxiety, and perceived lack of control are associated with uncooperative behavior in pediatric dental visits [5]. Shyness, a social–cognitive trait, affects how children approach unfamiliar situations. Shy children tend to be socially inhibited, which may cause passive resistance, reluctance to follow instructions, or reduced communication with the dentist [3,12]. Some studies show shy children may comply with treatment under social pressure, while others report withdrawal or refusal [4]. Although data on shyness in Saudi pediatric dentistry is limited, its potential impact is significant, especially given the high rates of dental anxiety among Saudi children [8].

### Research Aim

This study aims to evaluate the cognitive and emotional determinants, specifically fear of losing control, shyness, self-consciousness about dental aesthetics, child dental anxiety, parental dental anxiety, and past dental experiences, that influence cooperative versus uncooperative behavior in Saudi pediatric dental patients. The findings will provide evidence-based strategies for behavioral management in pediatric dentistry in Saudi Arabia.

## 2. Subjects and Methods

### 2.1. Study Design, Setting, and Ethical Approvals

A cross-sectional methodology was adopted to examine the dental profiles of children aged 6–11 years in Jeddah, Saudi Arabia. Data collection spanned December 2022 through November 2023 and included participants from King Abdulaziz University Dental Hospital (UDH), Ministry of Health (MOH) facilities (King Fahad General Hospital and North Jeddah Speciality Dental Center), King Fahad Armed Forces Hospital (KFAFH), and King Abdulaziz Medical City (KAMC). The study was conducted in accordance with the Strengthening the Reporting of Observational Studies in Epidemiology (STROBE) guidelines. Ethical approvals were obtained from both the Research Ethics Committees of the Faculty of Dentistry at King Abdulaziz University, Faculty of Dentistry (KAUFD) (162-12-22) and the National Guard at King Abdullah International Medical Research Center (1779/23). Written informed consent was obtained from all parents or legal guardians prior to participation.

### 2.2. Sample Size Estimation

The required sample size was calculated using OpenEpi Version 3.01, referencing findings by Herhaus et al. on anxiety and health status across varying BMI classifications [15]. With a mean difference of 0.59, 80% power, and a 95% confidence interval, the total sample size recommendation was 870 participants.

### 2.3. Participant Recruitment and Eligibility

Participants were consecutively recruited during the study period across the participating clinical sites. All eligible children who met the inclusion criteria were invited to participate, resulting in a final analytic sample of 906 children. Children aged 6–11 years attending dental clinics in Jeddah, Saudi Arabia, were considered eligible. The study sample was derived from the same cohort previously described in our earlier cross-sectional investigation examining BMI and dental anxiety [16]. Inclusion criteria were: (i) children aged 6–11 years, with no history of invasive dental treatment requiring local anesthesia; (ii) attendance for non-invasive dental procedures not requiring local anesthesia; and (iii) ability to complete behavioral and anxiety assessments using validated instruments, such as the Abeer Children Dental Anxiety Scale (ACDAS) and the Frankl scale. Exclusion criteria were: (i) children requiring emergency dental treatment, (ii) those with uncontrolled systemic or developmental conditions that could affect behavioral assessment, and (iii) inability to provide reliable responses during assessment.

### 2.4. Data Collection Tools and Measurements

Data were collected through structured interviews with child–parent pairs immediately following dental procedures. Variables included demographic characteristics, child cognitive responses, and parental dental anxiety, assessed using validated instruments (ACDAS and Frankl scale). Demographic data on the child’s gender, age grouping (10–11, 8–9, or 6–7), and household income were recorded. Income levels were classified as low (<7000 SAR), moderate (7000–12,000 SAR), or high (>12,000 SAR), based on regional socioeconomic categorizations [17]. For international comparability, approximate equivalents in US dollars (USD) were calculated using the prevailing exchange rate at the time of analysis (1 SAR ≈ 0.27 USD). The ACDAS consists of 13 child self-report items, three cognitive-focused questions, and three parent–dentist observations. The first 13 items yield a total score between 13 and 39, with values of 26 or higher denoting elevated anxiety [18]. All participants underwent standardized non-invasive procedures (basic prophylaxis and fluoride application), performed under consistent clinical conditions, including similar operatory settings, trained clinicians, and caregiver presence. Frankel’s classification of behavioral responses in the dental setting labels responses as definitely negative, negative, positive, or definitely positive. Children rated as negative or definitely negative are regarded as uncooperative, whereas those classified as positive or definitely positive are considered cooperative. Both the ACDAS and Frankel’s classification were implemented immediately after a basic prophylaxis and fluoride treatment. A preliminary assessment was conducted with 10 children not included in the main study to establish face validity, resulting in a content validity index (CVI) of 0.98. Moreover, the internal reliability of the ACDAS was confirmed, indicated by a Cronbach’s alpha of 0.91.

### 2.5. Statistical Analysis

All analyses were conducted using IBM SPSS Statistics 29.0. First, Chi-square tests were performed to assess associations between categorical variables and children’s cooperation status (Frankl 1–2 vs. 3–4). Variables analyzed included shyness in the clinic, shyness about teeth appearance, and fear of losing control. Next, independent samples *t*-tests were applied to compare BMI and dental anxiety scores (child and parental) between the cooperative and uncooperative groups. Finally, Multivariable logistic regression analysis was conducted to identify independent predictors of cooperation. The dependent variable was cooperative behavior (coded as 1), and independent variables included demographic factors (gender, age, income), cognitive factors (fear of losing control and shyness in the clinic), and parental dental anxiety scores. All tests were two-tailed, and a *p*-value of <0.05 was set as the threshold for statistical significance.

The outcome was cooperation, coded from Frankl ratings as uncooperative (1/2) = 0 and cooperative (3/4) = 1. Categorical predictors (child shy in the clinic, shy about teeth appearance, and fear of losing control) were screened with Pearson’s chi-square. Continuous predictors (child dental anxiety, maternal dental anxiety, and paternal dental anxiety) were compared between cooperation groups using independent-samples *t*-tests (reporting mean differences and 95% CIs); when normality or variance equality was not met, we verified results with Mann–Whitney U or Welch’s t, respectively. Demographic descriptors (parental education, income, child’s school level, age group, sex) were tested bivariately with chi-square.

A multivariable binary logistic regression model was fitted with cooperative behavior (1) versus uncooperative behavior (0) as the dependent variable. Predictors entered simultaneously included gender, age, income, fear of losing control, shyness in the clinic, maternal dental anxiety, and paternal dental anxiety. Model diagnostics included tolerance/VIF to check multicollinearity (all VIFs ≈ 1.1–1.2), and the omnibus model χ^2^ to confirm overall fit (χ^2^ ≈ 228.9, *p* < 0.001). All tests were two-tailed with α = 0.05. Analyses used standard statistical software; cases with missing data on a given test were handled listwise for that analysis. The mother’s anxiety had 158 missing cases, and the father’s had 168 missing. Those cases were excluded from the relevant analyses only. No imputation procedures were applied. The logistic regression used listwise deletion across all predictors, the analytic sample for the multivariable model was N = 738, whereas bivariate chi-square analyses used all valid cases (N = 906).

## 3. Results

### 3.1. Participant Characteristics

A total of 906 children aged 6 to 11 years were included in the analysis. Based on the Frankl classification, participants were categorized as cooperative or uncooperative. The sample was relatively balanced by gender, with 418 males and 488 females. Across age groups, 226 children were aged 10 to 11 years, 301 were aged 8 to 9 years, and 379 were aged 6 to 7 years. Most parents had a university-level education or higher. The largest proportion of families reported a monthly income greater than 10,000 SAR, followed by those in the 5000 to 10,000 SAR category, and then those earning less than 5000 SAR. Overall, the distribution of demographic characteristics was similar across cooperative and uncooperative groups, as shown in Table 1.

### 3.2. Demographic Factors and Cooperative Behavior

Demographic characteristics did not differ meaningfully between cooperative and uncooperative children, Table 1. The distribution of gender, age group, parental education level, and household income was broadly comparable across both groups. Although minor variations were observed, such as a slightly higher proportion of uncooperative behavior among females, these differences were small and did not indicate a consistent pattern.

### 3.3. Cognitive and Emotional Factors

Differences in cognitive and emotional characteristics between cooperative and uncooperative children are presented in Table 2 and Table 3. Fear of losing control demonstrated the most pronounced difference between groups. A markedly higher proportion of children who reported fear of losing control were classified as uncooperative compared to those who did not report this fear (61.6% vs. 21.6%). Children who reported shyness in the clinic were also more likely to be uncooperative than those who did not report shyness (48.6% vs. 28.8%). In contrast, self-consciousness related to dental appearance showed only minimal differences between groups (39.0% vs. 31.7%). With respect to dental anxiety, uncooperative children exhibited substantially higher child dental anxiety scores compared to cooperative children (mean: 31.57 vs. 20.38). Maternal dental anxiety was also higher among uncooperative children (mean: 9.23 vs. 8.16), whereas paternal dental anxiety showed only a modest difference between groups (mean: 7.15 vs. 6.69) as seen in Table 3.

### 3.4. Multivariable Logistic Regression Analysis of Determinants of Cooperative Behavior

The results of the multivariable logistic regression analysis are presented in Table 4. Fear of losing control emerged as the strongest independent determinant of cooperative behavior. Children who reported fear of losing control were significantly less likely to exhibit cooperative behavior during dental treatment (AOR = 0.14, 95% CI: 0.10–0.22). Maternal dental anxiety was also independently associated with cooperative behavior, with higher levels corresponding to a reduced likelihood of cooperation (AOR = 0.96, 95% CI: 0.93–1.00). In contrast, gender, age, household income, shyness in the clinic, and paternal dental anxiety were not significantly associated with cooperative behavior after adjustment for other variables. The overall model was statistically significant and demonstrated acceptable explanatory power and fit, indicating that the included variables collectively contributed to the prediction of cooperative behavior.

## 4. Discussion

The findings of this study indicate that emotional and familial variables were more closely associated with pediatric dental behavior than demographic characteristics. In this sample of 906 children, parental education, income, age, gender, and child educational level were not significantly related to cooperative behavior. This pattern is consistent with previous literature suggesting that demographic variables demonstrate limited or inconsistent associations with dental anxiety and behavior when compared with psychological correlates [15]. Within this relatively homogeneous population, emotional and cognitive factors appeared to have greater explanatory relevance, in line with findings reported in Saudi pediatric contexts [19].

Fear of losing control emerged as the most significant predictor of uncooperative behavior. This construct reflects the child’s perception of limited agency during dental procedures, which may contribute to heightened emotional distress and behavioral resistance. These results are consistent with the Cognitive-Affective Model of Dental Anxiety, which proposes that anxiety arises from cognitive appraisals of uncontrollability and threat [13]. According to this framework, perceptions such as an inability to influence events may trigger fear responses that manifest behaviorally.

The findings may also be interpreted within the framework of Locus of Control Theory. Rotter [20] proposed that individuals with a more external locus of control may be more vulnerable to anxiety in situations perceived as unpredictable or uncontrollable. In pediatric dental settings, perceived lack of control may therefore contribute to distress and behavioral difficulty. Empirical evidence indicates that providing children with limited procedural choices (e.g., selecting a fluoride varnish flavor) can improve behavioral responses during dental visits [21]. In addition, research in Saudi pediatric populations has reported associations between family context (e.g., siblings/birth order) and children’s dental anxiety and behavior [22]. Collectively, these findings suggest that perceived control and related cognitive appraisals represent relevant dimensions to consider in pediatric dental behavior management.

Parental factors were also associated with child behavior. Maternal dental anxiety remained significantly associated with child behavioral outcomes in multivariable analysis, whereas paternal dental anxiety was not independently associated after adjustment. This pattern is consistent with evidence indicating associations between parental and child dental fear [6,23], potentially reflecting modeling or observational learning processes. Recent research in Saudi pediatric settings has also examined parental anxiety and coping characteristics in relation to children’s behavior during dental procedures, including under moderate sedation [24]. These findings highlight the importance of considering parental psychological factors when planning pediatric dental care.

Although fear of pain and procedural discomfort are commonly emphasized in pediatric dentistry, the present findings suggest that perceptions of uncontrollability may represent a central cognitive vulnerability. Procedures such as injections or drilling may be experienced as distressing when perceived as unavoidable or unmanageable. Behavioral management strategies that incorporate structured choice, clear communication, and child-centered approaches may therefore address the cognitive aspects of anxiety more directly.

Recent evidence further supports the central role of psychological and cognitive determinants in pediatric dental behavior. A recent systematic review demonstrated that parental dental anxiety and psychological variables are consistently stronger predictors of children’s dental fear than demographic characteristics [25]. Additionally, newer systematic reviews and meta-analyses have emphasized the importance of behavioral and cognitive management strategies in enhancing cooperation. For instance, nonpharmacological behavioral interventions that improve predictability and increase patient engagement have been shown to significantly reduce dental anxiety in children [26]. Furthermore, family-related factors continue to play a central role. Parental dental anxiety remains a significant contributor to children’s behavioral responses, supporting the relevance of familial influences [27]. Collectively, these findings reinforce the importance of shifting from traditional demographic-based models toward psychologically informed approaches that emphasize cognitive vulnerability and family context. The clinical implications of these findings relate to the integration of psychological considerations into pediatric dental practice. Contemporary evidence shows that behavioral and cognitive strategies addressing fear and anxiety can significantly enhance patient cooperation and treatment results [28]. Identifying control-related anxiety and parental dental anxiety helps clinicians customize behavior management approaches. Screening tools and preparatory communication strategies support personalized care planning and may further improve cooperation during treatment. Clinical approaches that address children’s perceptions of control and maternal dental anxiety may therefore contribute to improved behavioral responses during treatment. Incorporating psychologically informed behavior management strategies into pediatric dental practice may support more effective and individualized care.

These findings support an evolving paradigm in pediatric dentistry, where behavioral management is increasingly guided by psychological profiling rather than demographic categorization. Recent evidence suggests that interventions targeting perceived control, communication, and emotional regulation are more effective in improving cooperation than approaches based solely on demographic risk factors [26,27]. This perspective aligns with contemporary patient-centered care models and highlights the importance of integrating both child and parental psychological characteristics into clinical decision-making.

This study has several limitations that should be considered when interpreting the findings. First, the cross-sectional design prevents making causal inferences, so the observed associations should be seen as correlations rather than cause-and-effect relationships. Second, behavioral and anxiety measures were partly based on self-reports and observational assessments, which could be affected by reporting bias or observer bias. Third, while important psychological and family variables were examined, other relevant factors, such as parenting style and child temperament and the qualitative nature of prior dental experiences (e.g., negative or traumatic encounters), were not fully explored and might have influenced the behavioral outcomes. Finally, since the study was conducted in a single geographic region in Saudi Arabia, the results may not be directly applicable to other populations or healthcare settings. Despite these limitations, the relatively large sample size and use of validated assessment tools support the reliability of the findings.

## 5. Conclusions

This study highlights fear of losing control and maternal dental anxiety as key predictors of children’s uncooperative behavior during dental visits, while demographic factors like age, gender, income, and education are insignificant. These findings shift the focus from traditional demographic profiling to addressing psychological and emotional readiness. Interventions that boost perceived control and reduce parental fear can significantly improve pediatric dental outcomes. Future strategies should incorporate these psychological insights to build trust, lessen anxiety, and enhance cooperation.

## Figures and Tables

**Table 1:** Participants’ demographic characteristics by Frankl Classification (cooperative vs. uncooperative).

Variable	Uncooperative (n, %)	Cooperative (n, %)	*p*-Value
**Father’s Education Level**			0.139
Elementary or less	8 (23.5%)	26 (76.5%)	
Middle/High School	131 (36.9%)	224 (63.1%)	
University or Higher	165 (31.9%)	352 (68.1%)	
**Mother’s Education Level**			0.589
Elementary or less	13 (37.1%)	22 (62.9%)	
Middle/High School	107 (31.6%)	232 (68.4%)	
University or Higher	184 (34.6%)	348 (65.4%)	
**Income**			0.608
Less than 5000 SR	26 (38.8%)	41 (61.2%)	
5000–10,000 SR	139 (33.7%)	274 (66.3%)	
More than 10,000 SR	139 (32.6%)	287 (67.4%)	
**Gender**			0.148
Male	130 (31.1%)	288 (68.9%)	
Female	174 (35.7%)	314 (64.3%)	
**Age Group**			0.182
10–11 years	66 (29.2%)	160 (70.8%)	
8–9 years	111 (36.9%)	190 (63.1%)	
6–7 years	127 (33.5%)	252 (66.5%)	
**Child’s Education Level**			0.710
Illiterate	27 (32.9%)	55 (67.1%)	
Elementary School	263 (33.3%)	526 (66.7%)	
Middle School	14 (40.0%)	21 (60.0%)	

Values are presented as frequency (percentage). Group differences were assessed using Pearson’s chi-square test. *p* < 0.05 considered statistically significant.

**Table 2:** Association between cognitive and emotional factors and cooperative behavior using Pearson’s chi-square test.

Variable	Uncooperative n (%)	Cooperative n (%)	χ^2^ (df = 1)	*p*-Value
**Shy in the clinic**			25.74	<0.001 *
No	209 (28.8%)	518 (71.2%)		
Yes	87 (48.6%)	92 (51.4%)		
**Shy about teeth appearance**			2.61	0.106
No	248 (31.7%)	535 (68.3%)		
Yes	48 (39.0%)	75 (61.0%)		
**Fear of losing control**			131.36	<0.001 *
No	142 (21.6%)	514 (78.4%)		
Yes	154 (61.6%)	96 (38.4%)		

* *p* < 0.05 considered statistically significant.

**Table 3:** Comparison of child and parental dental anxiety scores between cooperative and uncooperative groups using independent-samples *t*-tests.

Variable	Uncooperative (M ± SD)	Cooperative (M ± SD)	t (df)	*p*-Value	Cohen’s d
Child dental anxiety	31.57 ± 5.88	20.38 ± 5.87	26.91 (904)	<0.001 *	1.91
Maternal dental anxiety	9.23 ± 5.28	8.16 ± 4.90	2.73 (746)	0.006 *	0.21
Paternal dental anxiety	7.15 ± 4.11	6.69 ± 3.76	1.49 (736)	0.136	0.12

Results are presented as mean ± standard deviation. Analyses for parental anxiety scores were conducted using complete-case (listwise) deletion; sample size reflects available data. * *p* < 0.05 considered statistically significant.

**Table 4:** Multivariable Logistic Regression Analysis of Factors Associated with Cooperative Behavior (N = 738).

Variable	B	SE	*p*-Value	AOR (95% CI)
Gender	0.083	0.177	0.640	1.09 (0.77–1.54)
Age	0.005	0.043	0.903	1.01 (0.92–1.09)
Income	0.262	0.156	0.094	1.30 (0.96–1.77)
Fear of losing control	−1.939	0.204	<0.001 *	0.14 (0.10–0.22)
Shy in the clinic	−0.289	0.234	0.216	0.75 (0.47–1.18)
Maternal dental anxiety	−0.036	0.017	0.032 *	0.96 (0.93–1.00)
Paternal dental anxiety	−0.009	0.023	0.701	0.99 (0.95–1.04)

AOR = Adjusted Odds Ratio; CI = Confidence Interval. Model fit: Omnibus χ^2^(7) = 122.623, *p* < 0.001; Cox & Snell R^2^ = 0.153; Nagelkerke R^2^ = 0.214; Hosmer–Lemeshow χ^2^(8) = 8.856, *p* = 0.355; overall classification accuracy = 75.3%. * *p* < 0.05.

## Data Availability

The data supporting the findings of this study are available from the corresponding author upon reasonable request.

## Abbreviations

The following abbreviations are used in this manuscript:

KAUFD	King Abdulaziz University Faculty of Dentistry
STROBE	Strengthening The Reporting of Observational Studies in Epidemiology
VIF	Variance Inflation Factors
CVI	Content Validity Index
UDH	University Dental Hospital
MOH	Ministry of Health
KFAFH	King Fahad Armed Forces Hospital
KAMC	King Abdulaziz Medical Complex
ACDAS	Abeer Children Dental Anxiety Scale
